# Mechanisms of acclimation regulation and stress response to UV-B in *Dendrobium nobile* Lindl.

**DOI:** 10.3389/fpls.2026.1953265

**Published:** 2026-09-18

**Authors:** Jie Ma, Xuyang Liu, Yijun Fan, Li Chen, Mengjie Yuan, Chunhong Mou, Xiao Zhong, Meng Zhao, Haolin Liu, Junlan Wu, Jiaqing Liu, Yinxing Jiang, Yinglong Zhao, Aoxue Luo

**Affiliations:** Department of Landscape Plants, Sichuan Agricultural University, Chengdu, China

**Keywords:** acclimation responses, *Dendrobium nobile* Lindl., major chemical classes, regulatory mechanism, stress, UV-B

## Abstract

**Introduction:**

Ultraviolet-B radiation can enhance the accumulation of major chemical classes in medicinal plants but may simultaneously induce oxidative stress and cellular damage. Balancing these opposing effects is therefore critical for the effective application of UV-B in cultivation practices.

**Methods:**

In this study, *Dendrobium nobile* Lindl. plants were exposed to five different UV-B intensities (0, 20, 40, 80, and 160 μW cm-2) for 21 days. We systematically evaluated physiological parameters, measured the contents of major chemical classes (polyphenols, flavonoids, alkaloids, and polysaccharides), and performed transcriptomic profiling to investigate the underlying regulatory mechanisms.

**Results:**

Plants exhibited strong acclimation to 20 μW cm-2 UV-B, with contents of polyphenols, flavonoids, alkaloids, and polysaccharides increasing by 41.0%, 16.3%, 33.1%, and 19.3%, respectively (P < 0.05), while maintaining relatively low stress levels. Transcriptomic analysis revealed that at 20 μW cm⁻², transcription factors including *DnHY5, DnPCL1, DnMYBx1, DnBIM2, DnSCL9-l, DnMYBS3, DnBHLH66-l, DnWRKY2, DnPCF2, DnC3H2*, and *DnC3H14* may be involved in photomorphogenic acclimation, with key biosynthetic genes (*DnC4H, DnCAD, DnCHI, DnCHS, DnPOD12, DnMVK, DnMVD*) upregulated. In contrast, 80 μW cm-2 UV-B induced evident stress damage and activated stress-responsive genes (*DnXTH, DnCYP77A6, DnCER1, DnCry-DASH, DnPHR, DnH64PHR*), while suppressing several chemical biosynthesis-related genes.

**Discussion:**

These findings demonstrate that low-intensity UV-B (20 μW cm-2) promotes major chemical classes accumulation through photomorphogenic acclimation without significant stress damage, whereas high-intensity UV-B (80 μW cm-2) induces non-specific stress responses. This study provides a mechanistic framework for strategic UV-B application to improve medicinal plant quality and suggests optimized cultivation protocols for high-quality *D. nobile* production.

## 1 Introduction

*Dendrobium nobile* Lindl., a perennial epiphytic orchid, possesses significant horticultural and medicinal value, and is recognized as a precious resource in traditional Chinese herb (Li et al., 2023). *D. nobile* is rich in polysaccharides, polyphenols, alkaloids, and flavonoids, with pharmacological activities encompassing anti-aging, hypoglycemic, neuroprotective, anti-inflammatory, and immunomodulatory effects. Furthermore, its extracts have demonstrated antitumor efficacy (Fan et al., 2023a; Zhang et al., 2023). Consequently, strategies to enhance the major chemical classes of *D. nobile* have garnered increasing attention.

Targeted doses of UV-B (280–320 nm) can stimulate beneficial physiological responses in plants, yielding fruits, vegetables, and herbs enriched with secondary metabolites (Schreiner et al., 2012). As understanding of UV-B effects has advanced, research focus has shifted toward identifying specific regulatory factors rather than merely characterizing general stress responses (Jenkins, 2009). However, UV-B-induced stress occurs concurrently with beneficial processes, resulting in a complex and dynamic pattern of physiological responses to UV-B in plants (Podolec et al., 2021). The interactions between these dynamic processes remain incompletely understood. Consequently, the balance between acclimation and stress-induced damage is often overlooked when applying UV-B under controlled conditions. This study therefore aimed to develop strategies for harnessing UV-B to enhance *D. nobile* quality while mitigating its stress effects, and to elucidate the underlying regulatory mechanisms.

The effects of UV-B on plants manifest through two pathways: specific and non-specific signaling. The specific signaling pathway initiates via UVR8 photoreceptor. Upon UV-B, the active monomer of UVR8 interacts with nuclear proteins and genes, regulating the expression of key regulators involved in plant development and defense (Yin and Ulm, 2017). Concurrently, non-specific signaling pathways are activated under UV-B exposure, leading to the initiation of generalized stress responses (Hideg et al., 2013). Both can trigger multiple physiological reactions in plants, influencing their metabolism, growth, and overall fitness.

UV-B radiation universally promotes flavonoid biosynthesis in plants, with these metabolites serving as key agents in UV-B resistance (Neugart and Bumke-Vogt, 2021; Pandey et al., 2021). This regulatory process involves the UVR8 signaling pathway, where UVR8-HY5 interaction modulates structural gene expression (e.g., *CHS*, *DER1*) (Zhao et al., 2020b), while UVR8 also enhances transcription factor binding to flavonoid pathway promoters (e.g., MYB13), collectively enhancing flavonoid accumulation (Qian et al., 2020). However, significant differences exist in the biosynthesis of other metabolites across varying UV-B intensities. Li et al. (2021) reported that polysaccharide content in plants subjected to a low-intensity UV-B was significantly higher compared to other treatments. Low-intensity UV-B also stimulates ATP biosynthesis and promotes the accumulation of indole alkaloids (Zhong et al., 2021). Excessive UV-B can induce aberrant chromosome segregation and DNA damage, thereby disrupting normal physiological processes and impairing plant growth and development (Wang et al., 2021a). Plants counteract UV-B overexposure through multiple mechanisms, including the biosynthesis of flavonoids, anthocyanins, non-enzymatic antioxidants (e.g., ascorbic acid), and enzymatic antioxidants (e.g., catalase, ascorbate peroxidase, and peroxidase) (Jaiswal et al., 2020). Elevated UV-B levels also lead to disruptions in metabolite synthesis. For instance, Reyes et al. (2019) observed that starch content in *L. meyenii* decreased under prolonged high-intensity UV-B. Similarly, carotenoids are highly sensitive to strong UV-B and often undergo degradation under high UV-B doses (Guidi et al., 2016).

The differential effects of low versus high UV-B intensities on physiology and bioactive accumulation in *D. nobile*, the activation of distinct transcriptional programs for acclimation versus stress, and the optimal regime for balancing growth and major chemical class accumulation remain unclear. We hypothesized that low-intensity UV-B promotes secondary metabolism without oxidative damage (acclimation), whereas high-intensity UV-B induces non-specific stress responses with metabolic disruption, with the transition expected to occur within the tested gradient (0, 20, 40, 80, 160 μW cm^-2^). To test this, we examined a full intensity gradient, with 20 and 80 μW cm^-2^ selected as representative doses for comparative transcriptomic analysis. Integrating physiological measurements, biochemical assays, and transcriptomics, we characterized dose-dependent responses, identified acclimation- versus stress-related networks, and determined the optimal regime for bioactive accumulation. This study provides a mechanistic framework for UV-B regulation of quality in *D. nobile* with implications for light-managed cultivation.

## 2 Materials and methods

### 2.1 Plant materials

Two-year-old *Dendrobium nobile* Lindl. plants, obtained from Sichuan Lianhua Biological Science and Technology Co., Ltd., were grown in plastic pots (13.5 cm height × 14.5 cm diameter) filled with pine bark and clay pellets (6:1, v/v) at the Experimental Teaching Base of Sichuan Agricultural University (30°41′N, 103°49′E). Plants were maintained at 20 ± 5 °C and 70 ± 5% relative humidity, watered daily with 100 mL water, and received no fertilizer during the experimental period.

### 2.2 Radiation condition

Plants were placed on five 1 m³ steel frames enclosed with black cloth. Each frame was equipped with white fluorescent lamps (Philips Master TL-D 58 W/840; 120 μmol m^-^² s^-^¹) on a 10 h photoperiod (8:00-18:00). UV-B lamps (Philips PL-L 36W/01/4P; 305–315 nm, λ_peak_ = 311 nm) were mounted directly above the plants, with the distance adjusted to achieve the required intensities and periodically checked to maintain consistency. UV-B intensity was measured with a radiometer (Optoelectronic Instrument Factory, Beijing Normal University, China).

### 2.3 Experiment design

The experiment comprised five treatment groups. A total of 45 pots were utilized, with nine pots assigned to each group and ten plants per pot. The number of UV-B lamps and the distance to the *D. nobile* plants were adjusted individually to deliver UV-B radiation intensities of 20 μW cm^-2^ (T1), 40 μW cm^-2^ (T2), 80 μW cm^-2^ (T3), and 160 μW cm^-2^ (T4) for 10 hours daily (from 8:00 a.m. to 6:00 p.m.). The control group (CG) received white light irradiation alone. After 21 days, the leaves and stems of *D. nobile* were harvested for evaluation and analysis, while the remaining fresh samples were rapidly frozen and stored at −80 °C. Three biological replicates and three technical replicates were conducted for each treatment group.

### 2.4 Measurement of physiological indicators

After 21 days, the uppermost leaves were collected for physiological assays. Soluble protein (SP) was determined by the Coomassie Brilliant Blue method (Bradford, 1976) after 0.1 M NaOH extraction, with absorbance at 595 nm. Ascorbic acid (ASA) was measured at 534 nm following TCA extraction and sequential addition of phosphate-ethanol, BP-ethanol, and FeCl_3_-ethanol solutions (Tono and Fujita, 1981). Soluble sugars (SS) and malondialdehyde (MDA) were assessed by the thiobarbituric acid method (Heath and Packer, 1968; Wang et al., 2013); absorbance was measured at 450 and 532 nm after 10% TCA extraction and reaction with 0.6% TBA. Superoxide dismutase (SOD) was measured by the NBT method (Aguilar, 1993), catalase (CAT) by the ultraviolet absorption method (Aebi, 1984), and peroxidase (POD) by the guaiacol method (Chance and Maehly, 1955). Photosynthetic pigments were extracted from 0.2 g fresh leaf tissue with 80% acetone in darkness for 24 h, repeated until tissue was colorless, pooled to 25 mL, and absorbance measured at 663, 645, and 470 nm. Chlorophyll a, chlorophyll b, and lutein contents were calculated following Lichtenthaler (1987).

### 2.5 Determination of major chemical classes

Polysaccharides, flavonoids, polyphenols, and alkaloids were extracted from fresh *D. nobile* stems and quantified spectrophotometrically following established protocols. Polysaccharides were determined by the phenol-sulfuric acid method at 490 nm (Fan et al., 2023b). Flavonoids were measured at 510 nm using the NaNO_2_-Al(NO_3_)_3_-NaOH colorimetric system (Pękal and Pyrzynska, 2014). Total polyphenols were assayed with Folin-Ciocalteu reagent at 760 nm (Singleton et al., 1999). Alkaloids were extracted with chloroform after ammoniation and quantified at 620 nm by a modified spectrophotometric method (Shamsa et al., 2008). All contents were calculated from corresponding standard curves.

### 2.6 Transcriptome analysis

Transcriptome sequencing was performed on nine samples (T1, T3, and CG, three biological replicates each) using the Illumina NovaSeq 6000 platform with the TruSeq™ RNA Sample Preparation Kit. Raw reads were assembled *de novo* with Trinity, and transcripts were functionally annotated against NR, Swiss-Prot, Pfam, COG, GO, and KEGG databases, with additional homology inference via NCBI non-redundant protein database. Differential expression analysis was performed based on read counts, and DEGs were identified with |log_2_FC| ≥ 1 and FDR < 0.05.

### 2.7 qRT-PCR

The relative expression of nine target genes (*DnC4H*, *DnCAD*, *DnCHI3*, *DnBZ1*, *DnINV*, *DnPYL8*, *DnCER1*, *DnXYL4*, *DnTPS9*) was quantified by qRT-PCR. Purified RNA was reverse-transcribed using a cDNA synthesis kit (Sangon Biotech Co., Ltd., Shanghai, China). Primers were validated by gel electrophoresis, and qPCR was performed with 2× SG Fast qPCR Master Mix (Sangon Biotech Co., Ltd., Shanghai, China) on an ABI 7500 real-time PCR system (Applied Biosystems, Carlsbad, CA, USA) under 40 cycles of 95 °C for 5 s, 55 °C for 30 s, and 72 °C for 40 s (Arya et al., 2005).

### 2.8 Data analysis

Statistical analyses were performed using SPSS 25.0 (IBM Corp., Armonk, NY, USA). Normality and homogeneity of variances were confirmed by Kolmogorov-Smirnov/Shapiro-Wilk and Levene’s tests (p > 0.05). Group differences were assessed by one-way ANOVA followed by Tukey’s HSD *post-hoc* test (α=0.05). PCA and correlation analyses were conducted using Origin 2022 (OriginLab Corp., Northampton, MA, USA). Figures were prepared with Origin 2022, Adobe Photoshop 2021 (Adobe Inc., San Jose, CA, USA), and Microsoft Excel 2010 (Microsoft Corp., Redmond, WA, USA).

## 3 Results

### 3.1 Changes in phenotype

As shown in **Figure 1a**, no morphological damage was observed in *Dendrobium nobile* Lindl. under 21 days of continuous supplemental irradiation at T1 and T2. In contrast, leaf injury spots developed after 10 days under T3. Persistent stress induced leaf yellowing and abscission from day 15 onward; however, subsequent UV-B exposure did not cause further expansion of existing lesions. At T4, UV-B stress effects were more severe, with plants exhibiting distinct necrotic spots by day 5. The quantity of fallen leaves was much larger than that of other treatments, and the damaged areas continued to expand with prolonged irradiation. These results demonstrate that *D. nobile* acclimates to UV-B below 40 μW cm^-^² without adverse morphological effects, whereas higher irradiation levels induced stress responses manifested through chlorosis, lesion formation, and leaf abscission.

**Figure 1:**
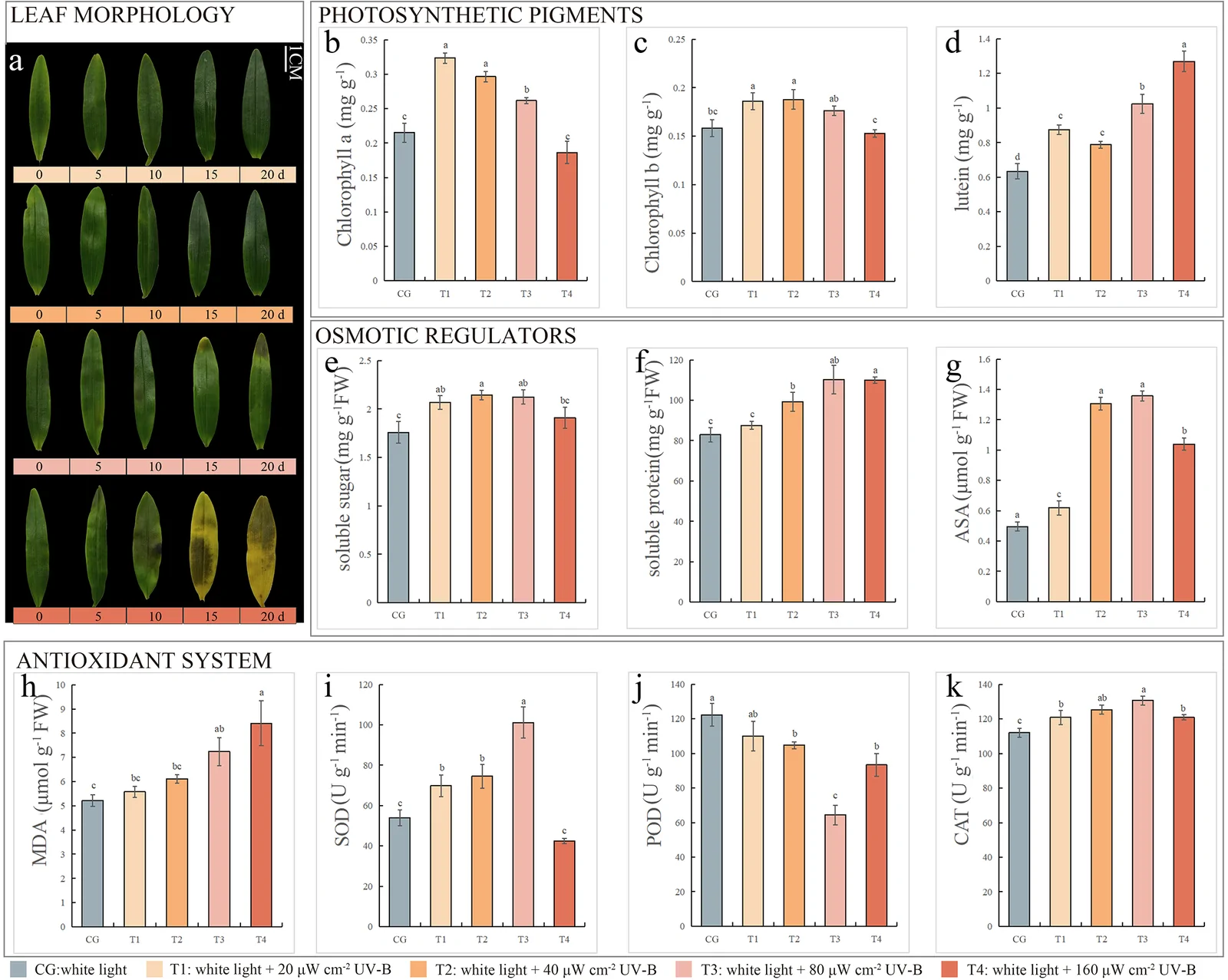
Morphological, physiological, and biochemical responses of *Dendrobium nobile* Lindl. to different UV-B radiation intensities over a 21-day period. **(a)** Temporal changes in leaf morphology at 5-day intervals. **(b)** Chlorophyll a content. **(c)** Chlorophyll b content. **(d)** Lutein content. **(e)** Soluble sugar content. **(f)** Soluble protein content. **(g)** Ascorbic acid content. **(h)** MDA content. **(i)** SOD activity. **(j)** POD activity. **(k)** CAT activity. Data are presented as mean ± SE (n = 3), with three biological replicates per group. Different letters above bars indicate significant differences among treatments (P < 0.05). Color codes: CG (gray), T1 (light yellow), T2 (yellow), T3 (pink), T4 (red).

### 3.2 Effects of different UV-B intensities on plant physiology and biochemistry

Chlorophyll a and b contents declined with increasing UV-B intensity across treatments, yet remained elevated in T1, T2, and T3 relative to CG after 21 days, with only T4 showing a decrease (**Figures 1b–d**). T1 demonstrated the highest chlorophyll a and b concentrations, exceeding CG by 50.5% and 16.9%, respectively. Conversely, T4 showed the lowest levels, representing reductions of 12.5% and 1.2% compared to CG. Lutein content in all treatments exceeded those in the CG, with T1 being 11.2% and 39.5% higher than T2 and the CG, respectively. From T2 to T4, lutein content increased with irradiation intensity, peaking in T4 at a level 103.7% above the CG. These findings indicate that low-intensity UV-B (20 μW cm^-^²) more effectively promoted chlorophyll accumulation, whereas high-intensity exposure (160 μW cm^-^²) impaired chlorophyll biosynthesis. Furthermore, all tested UV-B intensities generally elevated lutein content in *D. nobile.*

The effects of different UV-B intensities on three osmotic regulators (SS, SP, and ASA) displayed a parabolic response pattern (**Figures 1e–g**). SS content increased significantly under UV-B intensities below 80 μW cm^-^², but remained relatively stable across different intensities within this range. SP and ASA contents were significantly elevated in T2, T3, and T4 compared to CG and T1. ASA content showed substantial differences, with T2 and T3 demonstrating increases of 163.7% and 174.1%, respectively, relative to CG. These findings indicate that *D. nobile* acclimates to low-intensity UV-B, maintaining normal levels of osmoregulatory substances. In contrast, plants counteracted osmotic pressure changes induced by excessive UV-B intensity by sustainably increasing concentrations of osmoregulatory substances, particularly ASA and SP. However, excessively high UV-B ultimately impaired plant physiological function, thereby disrupting the biosynthesis of related compounds.

MDA content increased in a dose-dependent manner under UV-B irradiation (**Figure 1h**). When UV-B intensity exceeded 40 μW cm^-2^, MDA content increased significantly. Compared with CG, MDA in the T3 and T4 groups increased by 39.2% and 61.6%, respectively. In contrast, MDA levels in T1 and T2 showed no significant changes compared to CG. The progressive increase in MDA indicates intensified membrane lipid peroxidation with increasing UV-B intensity, while the absence of significant MDA changes at lower doses suggests maintained membrane integrity under these conditions.

UV-B intensity and SOD/CAT activities exhibited an overall parabolic trend (**Figures 1i, k**). The highest activities were observed in the T3 group, with SOD activity increasing by 87.3% and CAT by 15.3% relative to CG. This coordinated upregulation was consistent with the oxidative challenge indicated by MDA. However, compared to T3, SOD and CAT activities in the T4 group declined with increasing UV-B intensity, with only CAT activity remaining above the CG level. POD activity showed a declining trend across all treatment groups (**Figure 1j**), with the most pronounced decrease observed in the T3 group (47.7% reduction compared to CG). Given that POD catalytic function strictly depends on the availability of phenolic electron donors, its activity decline may be associated with insufficient net supply of phenolic metabolites under UV-B stress.

### 3.3 Analysis of major chemical classes

Exposure to T1, T2, and T3 increased polysaccharide content by 19.3%, 13.1%, and 5.4%, respectively (**Figure 2a**). T4 exhibited robust polysaccharide biosynthesis, resulting in a 134.0% content increase. Mild UV-B intensity (T1) markedly enhanced polyphenol levels by 41.0% relative to CG, whereas no substantial increases were observed under T2, T3, or T4 (**Figure 2b**). UV-B intensity significantly influenced flavonoid content (**Figure 2c**), with total flavonoid levels in T1 exceeding those in CG and T2 by 16.3% and 13.4%, respectively. Flavonoid content demonstrated an increasing trend with rising irradiation intensity, reaching 41.7% higher in T4 than CG. Alkaloid content showed a notable increase of 33.1% in T1 (**Figure 2d**), surpassing all other treatment groups. These findings suggest that low-level UV-B induces minimal stress while enhancing the accumulation of multiple major chemical classes in *D. nobile*.

**Figure 2:**
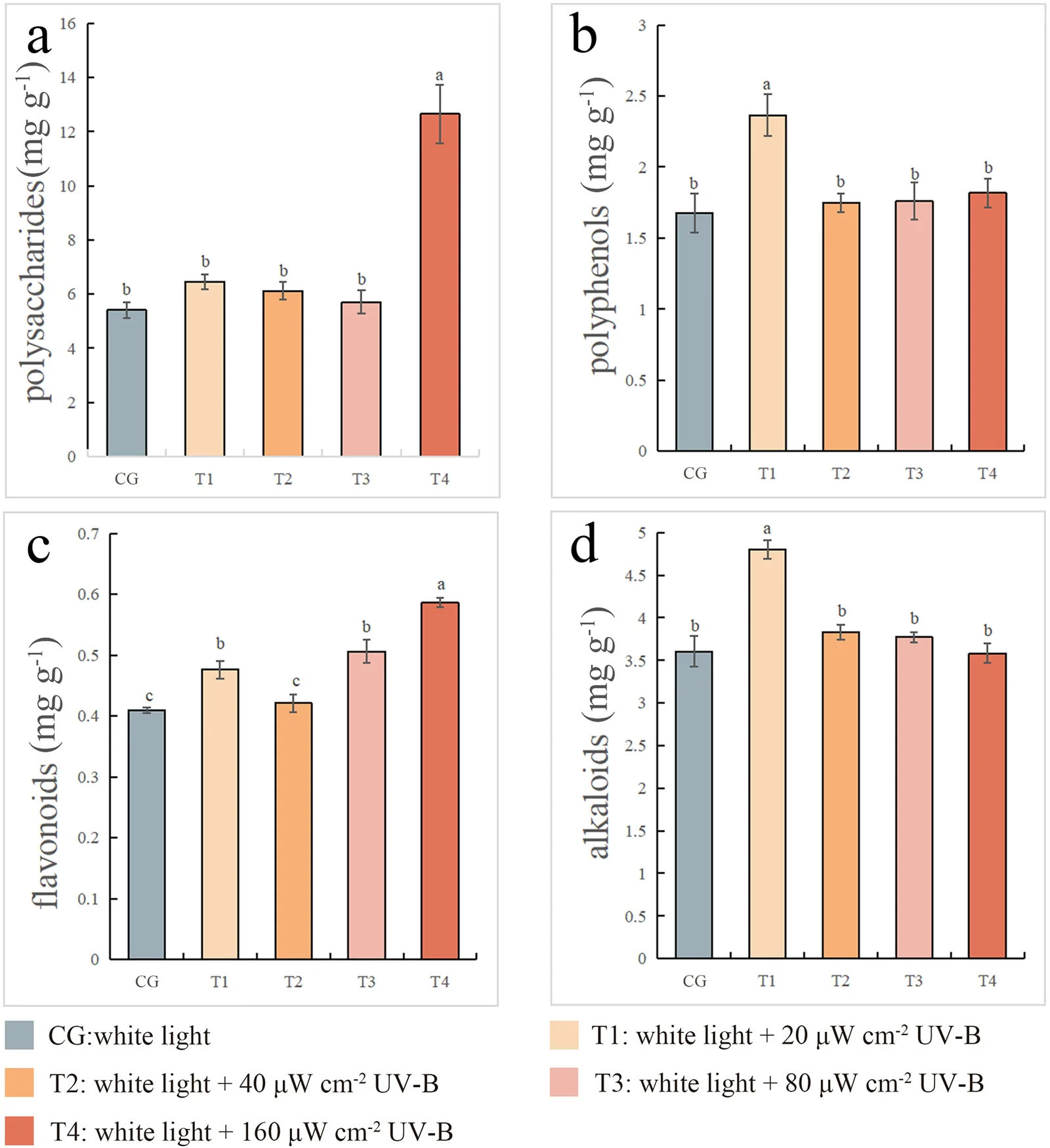
Effects of different UV-B radiation intensities on major chemical classes in *Dendrobium nobile* Lindl. **(a)** Polysaccharide content. **(b)** Polyphenol content. **(c)** Flavonoid content. **(d)** Total alkaloid content. Values are presented as mean ± SE (n = 3), with three biological replicates per group (minimum three plants each). Different letters above bars indicate significant differences among treatments (P < 0.05). Color codes: CG (gray), T1 (light yellow), T2 (yellow), T3 (pink), T4 (red).

### 3.4 Correlation analysis

**Figures 3a–d** presents correlation analyses between major chemical classes and physiological indices under increasing UV-B intensities versus the CG. Under T1, polysaccharides, flavonoids, polyphenols, and alkaloids all showed significant positive correlations with UV-B intensity. Polysaccharides also correlated positively with SS and ASA; polyphenols with SS and SOD; flavonoids with ASA and CAT; and alkaloids with SS, ASA, SOD, and CAT. In T2, only polysaccharides remained positively correlated with UV-B intensity, while SP, ASA, SOD, CAT, and SS were all strongly correlated with polysaccharide content. Under T3, flavonoids correlated positively with UV-B intensity, lutein, SS, ASA, and CAT. In T4, flavonoids and polysaccharides showed strong negative correlations with SOD and POD, but positive correlations with UV-B intensity, lutein, SP, ASA, and CAT. These results confirm that UV-B significantly modulates major chemical classes accumulation in *D. nobile*, with low-intensity irradiation (T1) most strongly associated with enhanced accumulation.

**Figure 3:**
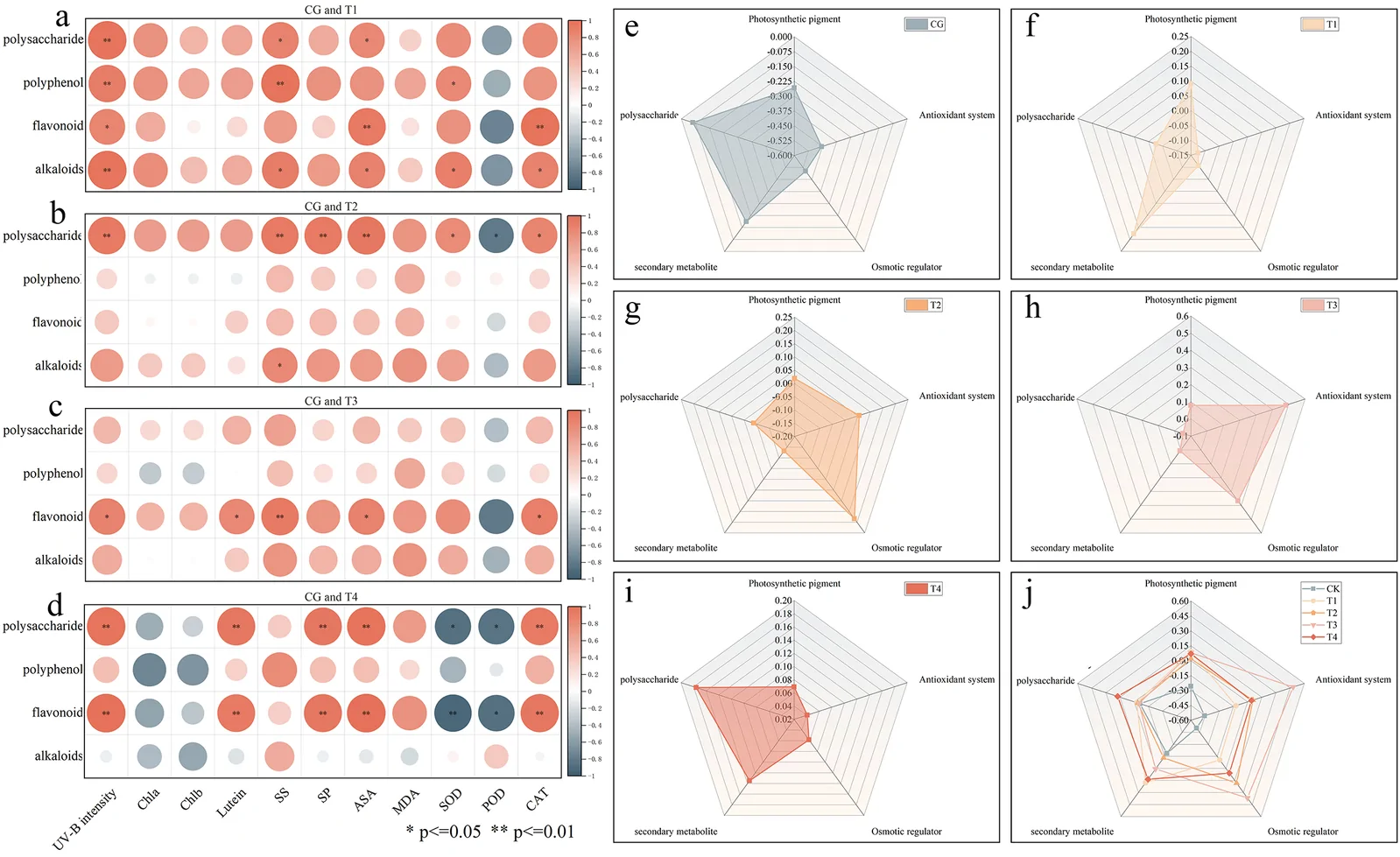
Correlation analysis of major chemical classes with variations in other indices across different UV-B radiation intensities, and schematic representation of stress regulation contributions by each index under varying UV-B intensities. **(a–d)** Correlation analysis of major chemical classes with other indicators in T1, T2, T3, and T4 treatment groups. **(e–i)** Schematic diagrams illustrating stress regulation contributions for CG, T1, T2, T3, and T4 groups; photosynthetic pigments include chlorophyll a, chlorophyll b, and lutein; antioxidant system components comprise MDA, SOD, POD, and CAT; osmotic regulators consist of soluble sugars (SS), soluble proteins (SP), and ascorbic acid (ASA); secondary metabolites encompass polyphenols, flavonoids, and alkaloids. **(j)** Comparative visualization of integrated stress regulation contributions across treatment groups. The radial axis length of each sector in the radar chart indicates the relative contribution of the corresponding category under each treatment condition. Sectors extending farther from the center represent greater functional importance. **Significant correlation at the 0.01 level; *Significant correlation at the 0.05 level.

### 3.5 Stress regulation contribution of each physiological resistance mechanism

The 14 physiological indices from five treatment groups (CG, T1, T2, T3, T4) underwent standardized processing followed by principal component analysis. Stress regulation contribution values were derived from each principal component’s contribution rate. All data were categorized into five resistance mechanisms: antioxidant system, photosynthetic pigments, secondary metabolites, polysaccharides, and osmoregulators. Contribution values for each mechanism were calculated, with radar charts generated to visualize treatment-specific patterns (**Figures 3e–i**). **Figure 3f** showed secondary metabolites and photosynthetic pigments dominating in T1. **Figures 3g, h** indicated antioxidant systems and osmoregulators predominating in T2 and T3, with antioxidant contribution increasing progressively with UV-B intensity. **Figure 3i** demonstrated markedly increased contributions of secondary metabolites and polysaccharides in T4. Comparative analysis (**Figure 3j**) revealed gradual increases in antioxidant systems and osmoregulators from CG to T3 with rising intensity, indicating enhanced antioxidant capacity. However, these contributions decreased significantly at T4, demonstrating impaired antioxidant function. Secondary metabolites showed highest contribution in T1, suggesting low UV-B intensity promotes their biosynthesis. Polysaccharides maintained similar contributions in CG, T1, T2, and T3 but increased significantly in T4, indicating enhanced biosynthesis when irradiation exceeded tolerance thresholds.

### 3.6 Principal component analysis

Principal component analysis was performed on two parameter sets: major chemical classes (polyphenols, polysaccharides, flavonoids, alkaloids) and stress resistance indices (SS, SP, ASA, MDA, POD, CAT, SOD). Following data standardization, a composite index F(x) was derived for each treatment via weighted summation. As shown in **Table 1**, T1 ranked highest in major chemical classes regulation (CG < T2 < T3 < T4 < T1), while **Table 2** shows T3 ranked highest in stress resistance (CG < T4 < T1 < T2 < T3). Overall, T1 exhibited enhanced secondary metabolism with lower antioxidant enzyme activities, indicating an acclimation response, whereas T3 showed maximal antioxidant engagement under significant UV-B stress. Notably, T4 displayed the most severe physiological damage and high accumulation of polysaccharides and flavonoids, yet ranked lower than T3 in stress resistance, suggesting passive accumulation rather than active defense. Based on this functional divergence, T1 (optimal acclimation) and T3 (peak active defense) were selected for transcriptomic analysis to elucidate their underlying regulatory mechanisms.

**Table 1:** Principal component analysis of the main major chemical classes components of *D. nobile* under different UV-B conditions.

Treatment	The score of the comprehensive index		Membership function value		D value	Rank
	F1	F2	U1	U2		
CG	-0.631	-0.939	0.000	0.000	0.000	5
T1	1.837	0.064	1.000	0.379	0.698	1
T2	-0.291	-0.691	0.138	0.094	0.116	4
T3	-0.295	-0.144	0.136	0.300	0.216	3
T4	-0.619	1.709	0.005	1.000	0.489	2
Weight			0.514	0.486		

F1 and F2 represent the scores of the first and second principal components, respectively. U1 and U2 are the membership function values derived from F1 and F2, indicating the relative performance of each treatment on each principal component (0 to 1 scale). The composite score (D) was calculated as the weighted sum: D = (U1 × 0.514) + (U2 × 0.486); T1 (20 μW cm^-2^), T2 (40 μW cm^-2^), T3 (80 μW cm^-2^), T4 (160 μW cm^-2^), CG (Control Group).

**Table 2:** Principal component analysis of the main resistance indexes of *D. nobile* under different UV-B conditions.

Treatment	A score of a comprehensive index		Membership function value		D value	Rank
	F1	F2	U1	U2		
CG	-1.457	-0.181	0.000	0.770	0.226	5
T1	-0.490	0.772	0.337	1.000	0.532	3
T2	0.451	0.378	0.665	0.847	0.718	2
T3	1.411	0.468	1.000	0.882	0.965	1
T4	0.085	-1.799	0.537	0.000	0.380	4
Weight			0.706	0.294		

F1 and F2 represent the scores on the first and second principal components, respectively. U1 and U2 are the membership function values derived from F1 and F2, normalized to a 0–1 scale. The composite score (D) was calculated as: D = (U1 × 0.706) + (U2 × 0.294); T1 (20 μW cm^-2^), T2 (40 μW cm^-2^), T3 (80 μW cm^-2^), T4 (160 μW cm^-2^), CG (Control Group).

### 3.7 Overview of DEGs

We performed transcriptome sequencing to examine UV-B intensity effects on *D. nobile*. Three treatments, CG, T1, and T3, were selected. Sequencing data analysis indicated Q20 >97.5%, Q30 >93.9%, and GC content >45.3%. Differentially expressed genes (DEGs) were identified among groups following differential expression analysis across treatments. Venn diagram analysis revealed that T1 and T3 exclusively regulated 240 and 998 genes, respectively, while sharing 167 common genes (**Figure 4a**). Volcano plot analysis showed 496 DEGs between T1 and CG (263 up, 233 down), 1,272 between T3 and CG (720 up, 552 down), and 390 between T3 and T1 (275 up, 115 down) (**Figures 4b-d**). Experimental data indicated that certain genes were consistently regulated under UV-B, irrespective of intensity. Moreover, as UV-B intensity increased, additional response mechanisms were activated in the plant, resulting in an increase in DEGs.

**Figure 4:**
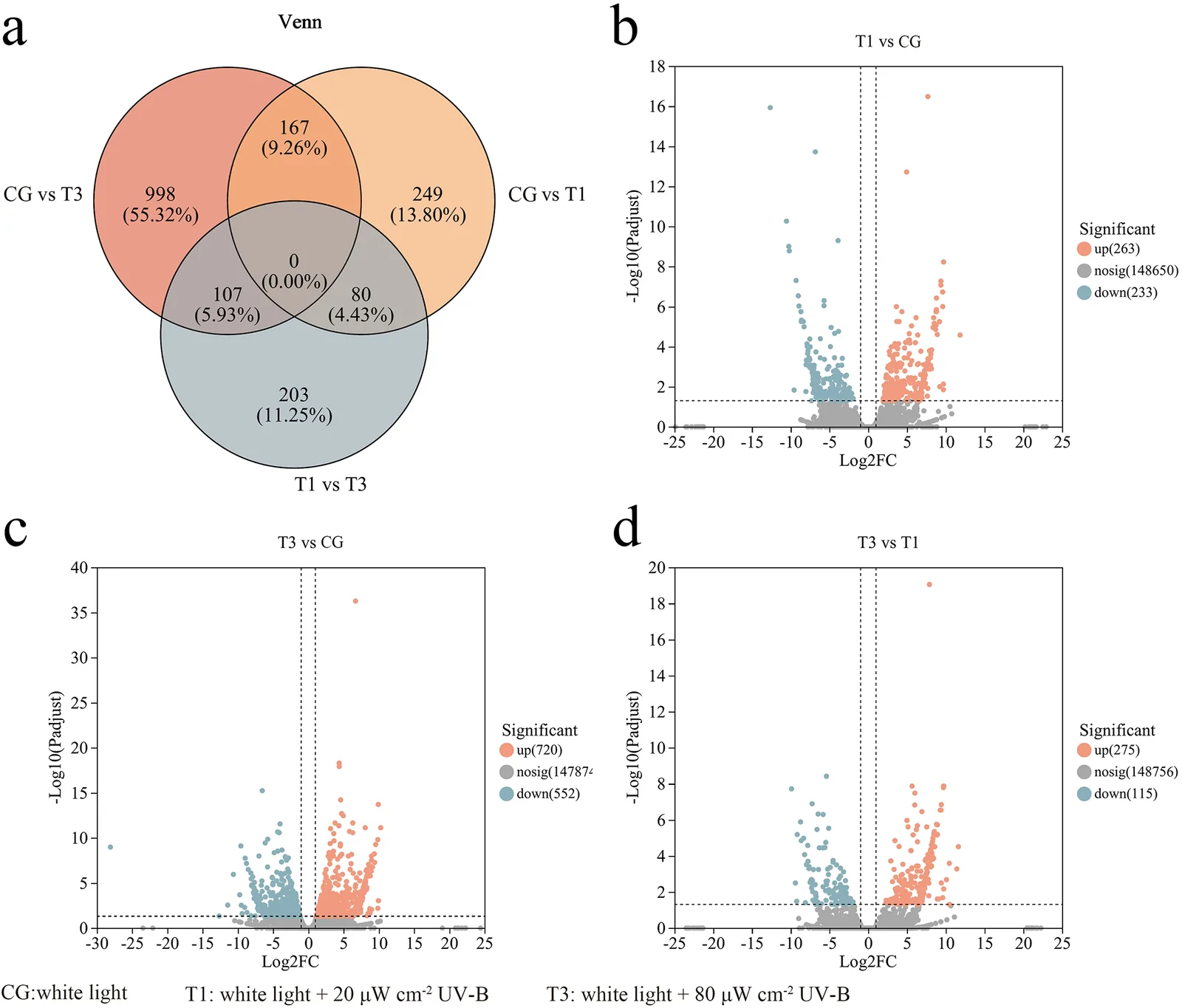
**(a)** Venn diagrams of overlap between DEGs in T1 vs CG, T3 vs CG, and T1 vs T3. **(b)** Differential gene volcano plots for T1 vs CG. **(c)** Differential gene volcano plots for T3 vs CG. **(d)** Differential gene volcano plots for T3 vs T1.

### 3.8 GO functional enrichment analysis

DEGs among groups were annotated against the Gene Ontology database (**Figure 5**). Both CG vs. T1 and CG vs. T3 showed enrichment in response to abiotic stimulus, light stimulus, and radiation. CG vs. T1 specifically enriched for ROS response, oxidative stress, temperature stimulus, heat, hydrogen peroxide, and cellular responses to radiation and light. CG vs. T3 enriched in red/far-red light response, extracellular stimulus, cell wall macromolecule metabolism, xyloglucan and hemicellulose metabolism, nutrient levels, and cell wall polysaccharide metabolism. Within photostimulation and radiation categories, genes including *DnGIL1-like*, *Dn(6-4)DNA photolyase*, *DnCOP1*, *DnsigD*, *DnHY5-like*, *DnSPA1*, *DnBIC1-like*, *DnLHCA3*, *DnHY5*, and *DnCat1* showed progressive upregulation with increasing UV-B intensity, while *DnB-box20-like* and *DnFT* were downregulated. Under 20 μW cm^-^² UV-B, *DnHSP16.0*, *DnsHsp*, *DnHSP17.4*, and *DnHSP17.6* were upregulated and *DnclpB3* downregulated in heat response. At 80 μW cm^-^², cell wall metabolism genes *DnXTH32*, *DnXTH9*, and *DnCLP1* were upregulated, while *DnXYL4*, *DnBXL7*, *DnXTH2*, and *DnXTH23* were downregulated.

**Figure 5:**
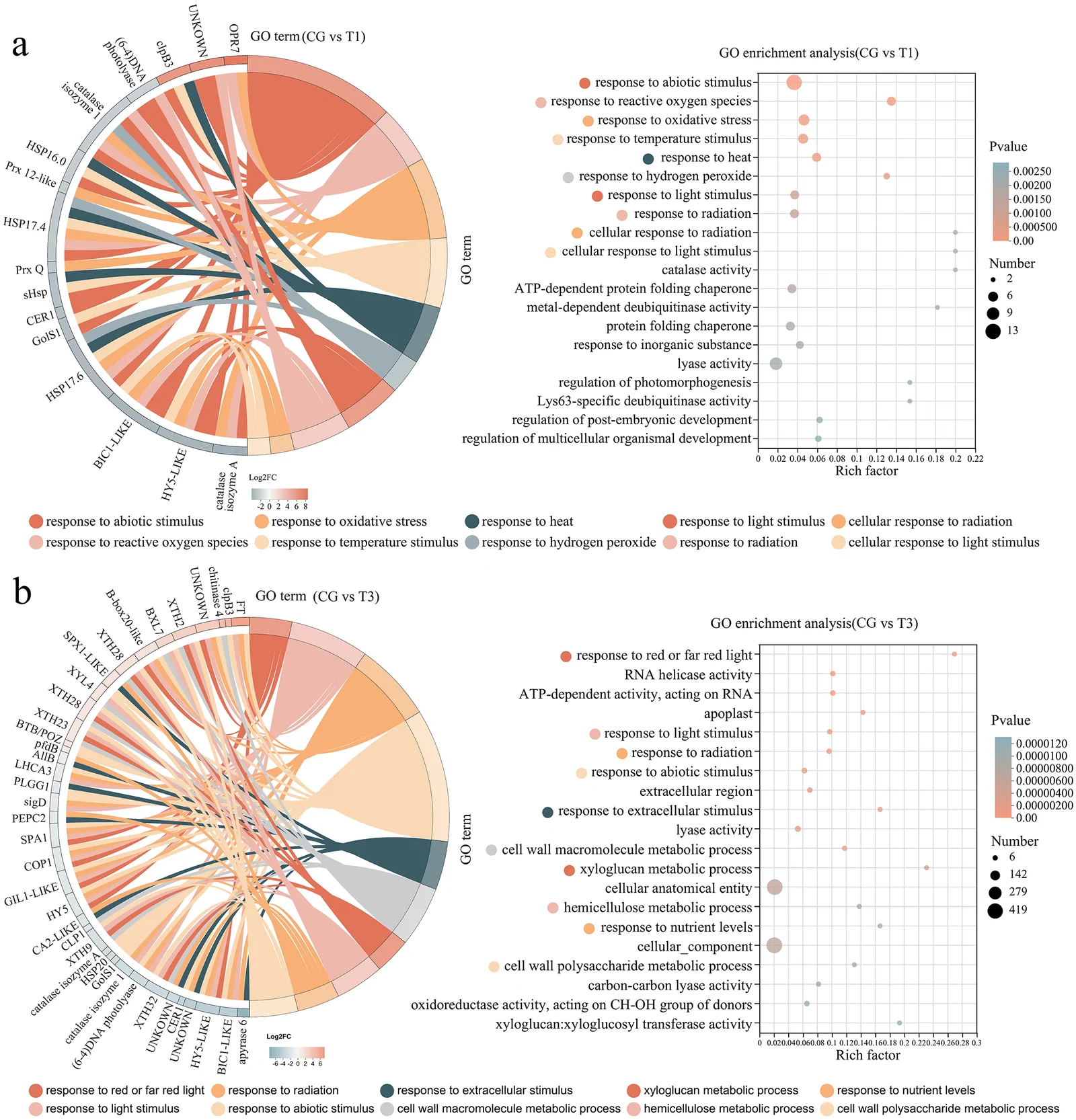
Functional enrichment analysis. **(a)** GO enrichment and GO chord plot analysis of CG vs T1. **(b)** GO enrichment and GO chord plot analysis of CG vs T3.

### 3.9 KEGG functional annotation and enrichment analysis

The DEGs were separately annotated against the KEGG database. As shown in **Figure 6**, the DEGs of *D. nobile* under T1 vs. CG were mainly annotated in carbohydrate metabolism, amino acid metabolism, and energy metabolism within metabolic processes; folding, sorting and degradation, and translation within genetic information processing; transport and catabolism within cellular processes; and signal transduction within environmental information processing. The DEGs in T3 vs. CG, in addition to a significant increase in annotation number for the same functional categories, included global and overview maps, glycine biosynthesis and metabolism, and nucleotide metabolism within metabolic pathway annotations. Comparison of KEGG enrichment analysis for T1 vs. CG revealed that the DEGs were mainly enriched in protein processing in the endoplasmic reticulum, glyoxylate and dicarboxylate metabolism, galactose metabolism, MAPK signaling pathway, and other glycan degradation. The DEGs of T3 vs. CG were enriched in carbon fixation in photosynthetic organisms, flavonoid biosynthesis, photosynthesis, glycerophospholipid metabolism, and anthocyanin biosynthesis. These results demonstrate that *D. nobile* responds to different intensities of UV-B by regulating diverse genes.

**Figure 6:**
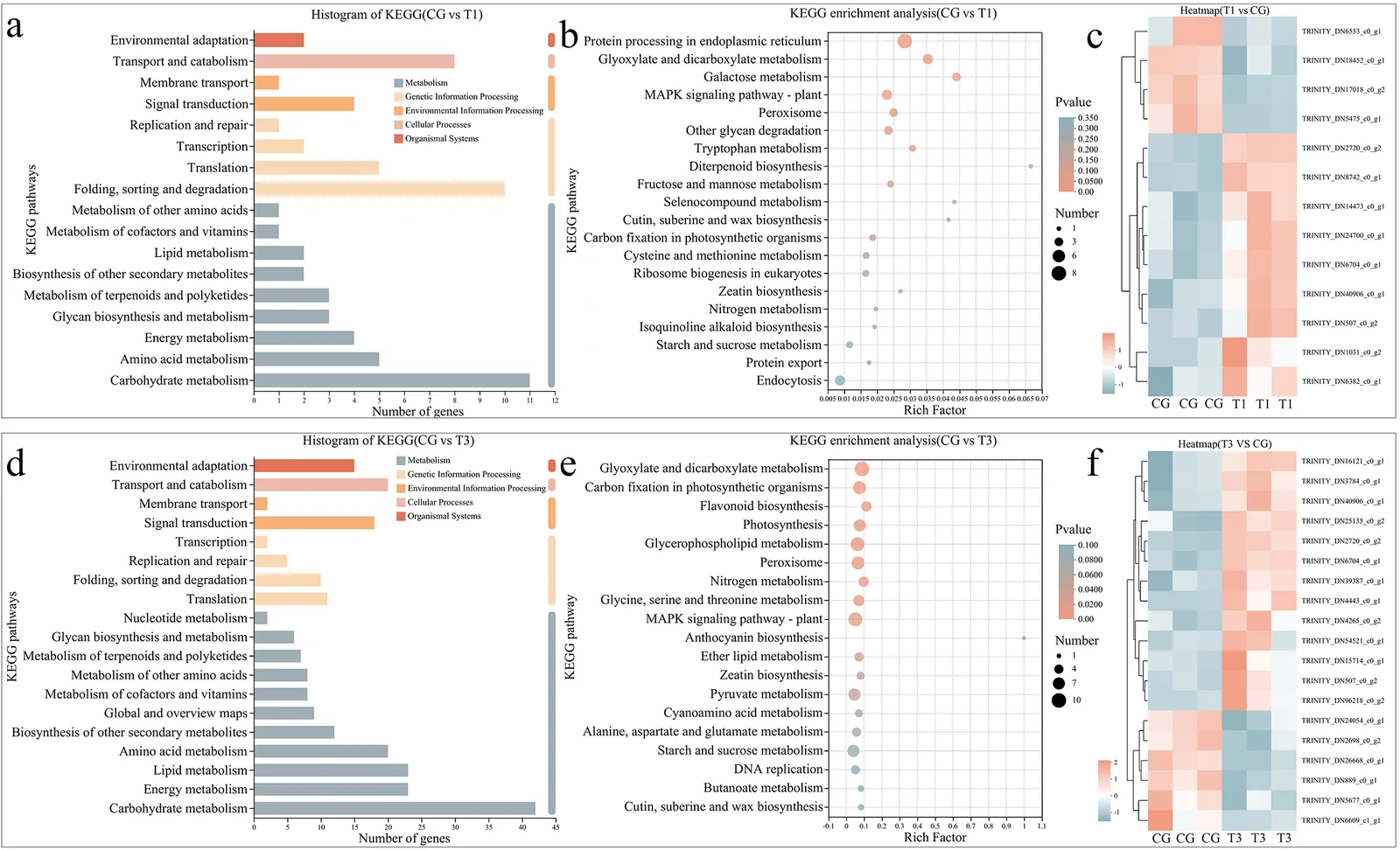
Analysis of KEGG. **(a)** KEGG annotation analysis of CG vs T1. **(b)** KEGG enrichment analysis of CG vs T1. **(c)** Analysis of major genes in KEGG enrichment of CG vs T1. **(d)** KEGG annotation analysis of CG vs T3. **(e)** KEGG enrichment analysis of CG vs T3. **(f)** Analysis of major genes in KEGG enrichment of CG vs T3.

### 3.10 Differentially expressed transcription factor analysis

As shown in **Figure 7a**, multiple transcription factor families were highly responsive to UV-B. The most abundantly annotated families included MYB, AP2/ERF, bHLH, NAC, C2C2, WRKY, and GRAS. Analysis of transcription factor expression profiles (**Figure 7b**) demonstrated that genes in the MYB, AP2/ERF, bHLH, WRKY, and GRAS families displayed considerable variation in expression under different UV-B intensities. These results indicated that a coordinated regulatory network was activated in *D. nobile* in response to UV-B, wherein multiple transcription factors collectively modulated functional gene expression across varying UV-B intensity levels.

**Figure 7:**
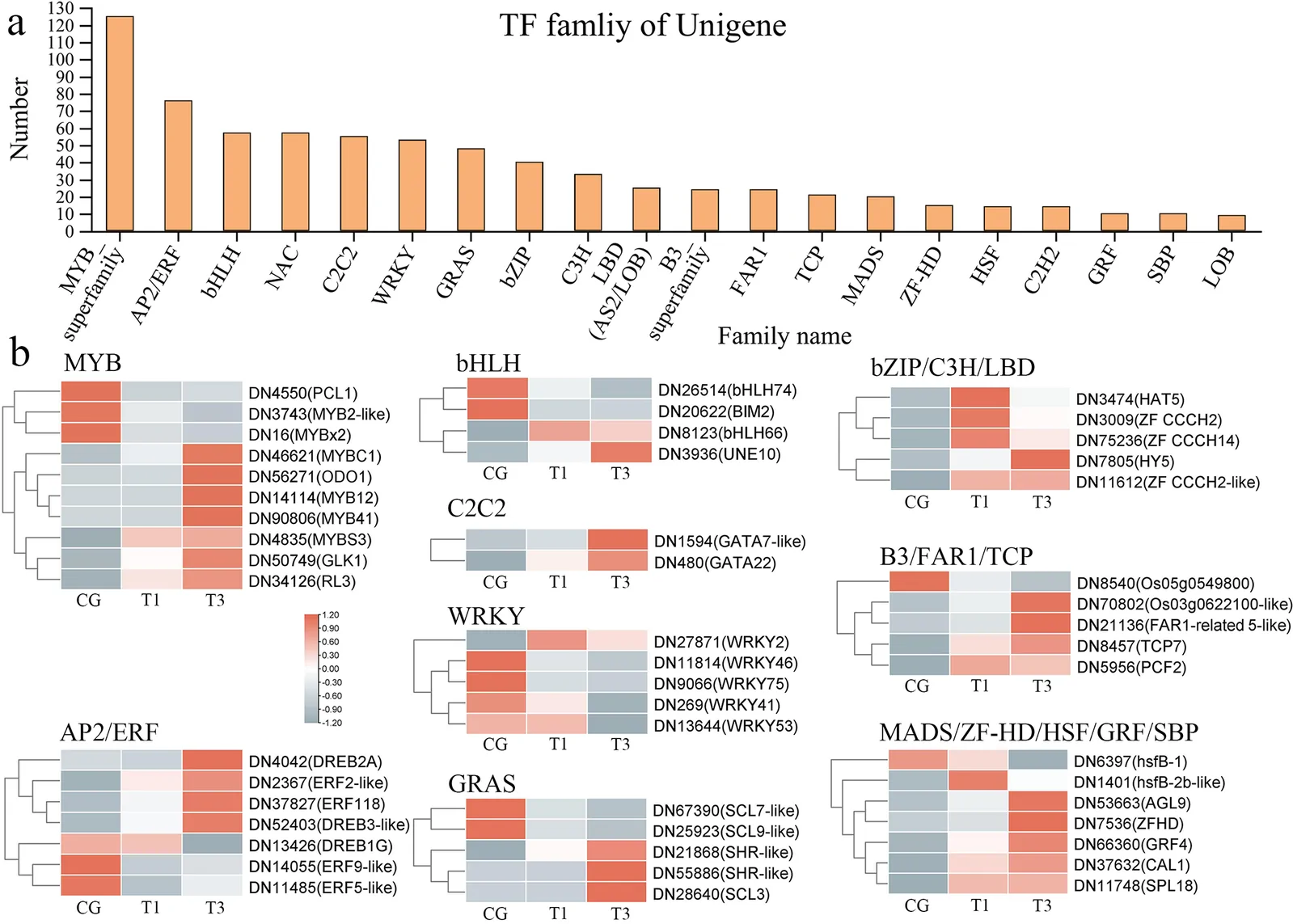
**(a)** Transcription factor family analysis. **(b)** Differential expression analysis of major transcription factor genes.

### 3.11 Key gene analysis

Multiple metabolic pathways showed differential gene regulation under UV-B stress (**Figure 8**). In phenylpropanoid biosynthesis, *DnC4H* was intensity-dependently upregulated, while *DnPOD12* and *DnCAD* showed higher expression in T1, and *DnBIA1* was downregulated (**Figure 8a**). Flavonoid biosynthesis involved upregulation of *DnC4H*, *DnCHS*, *DnCHI*, *DnCHI3*, and *DnBZ1* (involved in anthocyanin biosynthesis), with more pronounced upregulation in T3 (**Figure 8b**). Polysaccharide biosynthesis pathways responded differentially to UV-B intensities (**Figure 8c**). In starch and sucrose metabolism, *DnINV* and *DnTPP6* were upregulated in both groups, while *DngblB* and *DnTPS* were downregulated in T3. Fructose and mannose metabolism showed *DnGMPP* upregulated in both groups, *DnMAN6* and *DnMAN9* downregulated in T3 but upregulated in T1. Galactose metabolism exhibited *DngalM* downregulation and *DnINV/DnGOLS* upregulation in both treatments. Xylose metabolism showed *DnXYL4* downregulation only in T3. Terpenoid backbone biosynthesis primarily involved mevalonate pathway, with *DnMVD* upregulated in both groups, *DnMVK* upregulated in T1 but downregulated in T3, and *DnTPS9* upregulated in both treatments (**Figure 8d**). Chlorophyll degradation gene *DnSGR* and phytol synthesis gene *DnGGR* were downregulated (**Figure 8e**). Cuticle synthesis genes (*DnCYP77A6* and *DnCER1*) and photolyase genes (*DnH64PHR*, *DnPHR2*, *DnPHR1*, *DnCry-DASH*) were upregulated with escalating UV-B intensity (**Figures 8f, h**). ABA signaling showed *DnMAPKKK17* upregulated only in T3; *DnPYL3* and *DnPYL8* more strongly upregulated in T3; *DnSAPK2*, *DnSAPK7*, and *DnPP2C* upregulated in both groups (**Figure 8g**). UV-B signaling pathway genes (*DnUVR8*, *DnHY5-like*, *DnHY5*, *DnSPA1*, *DnCOP1*, *DnRUP2*) were upregulated, with all except *DnRUP2* showing intensity-dependent upregulation.

**Figure 8:**
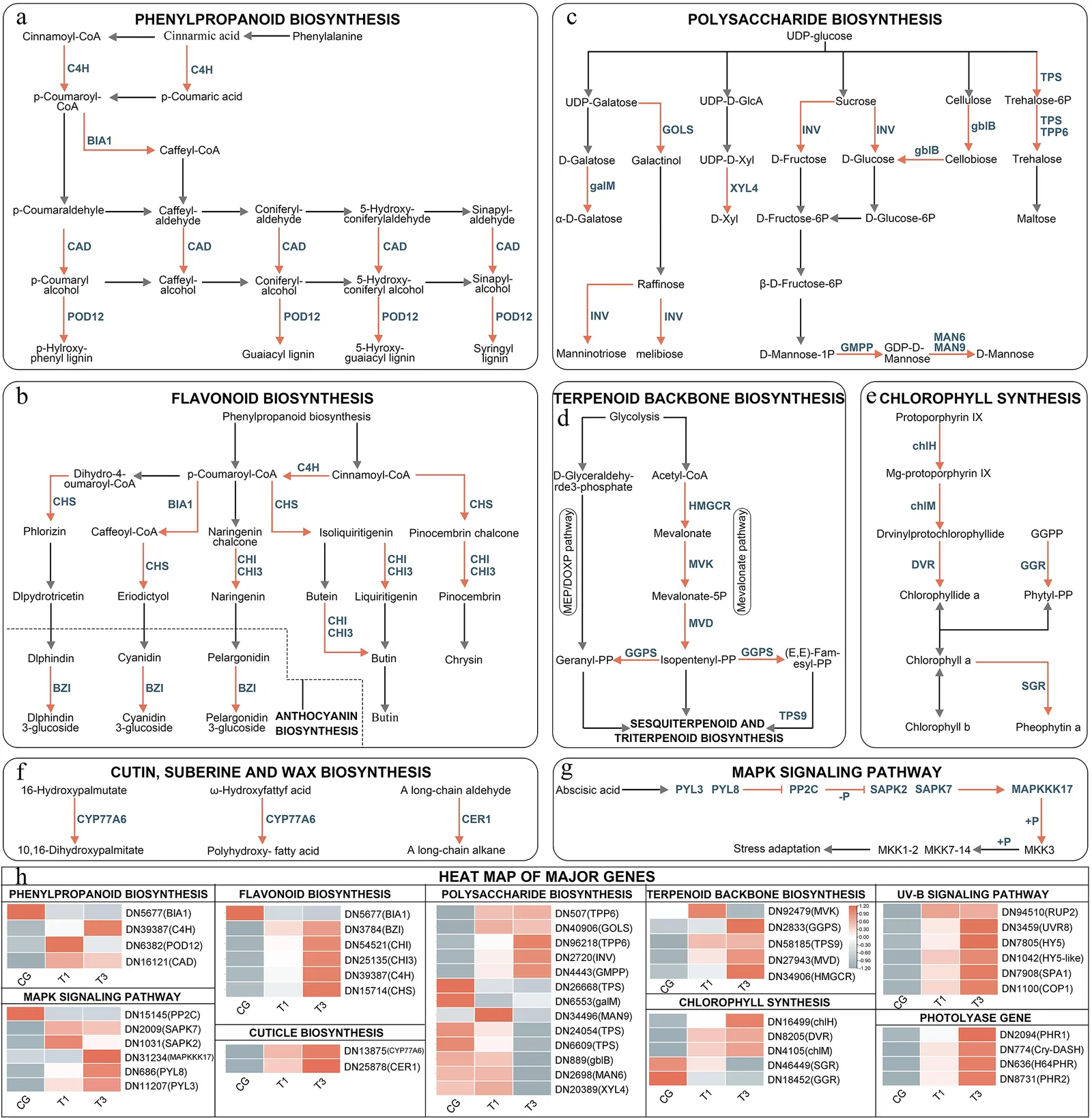
KEGG pathway analysis. **(a)** Is Phenylpropanoid biosynthesis. **(b)** Is Flavonoid biosynthesis. **(c)** Is Fructose and mannose metabolism, Starch and sucrose metabolism, Amino sugar and nucleotide sugar metabolism, and Galactose metabolism. **(d)** Is Terpenoid backbone biosynthesis. **(e)** Is Chlorophyll synthesis pathway. **(f)** Is cutin, suberine and wax biosynthesis. **(g)** Is MAPK signaling pathway. **(h)** Is the heat map of major genes.

### 3.12 qPCR validation results

Nine key DEGs (*DnC4H, DnCAD, DnCHI3, DnBZ1, DnINV, DnPYL8, DnCER1, DnXYL4, DnTPS9*) were randomly selected for validation by RT-qPCR. The expression trends of these genes were generally consistent with the transcriptomic data, confirming the reliability of the RNA-seq results (**Figure 9**).

**Figure 9:**
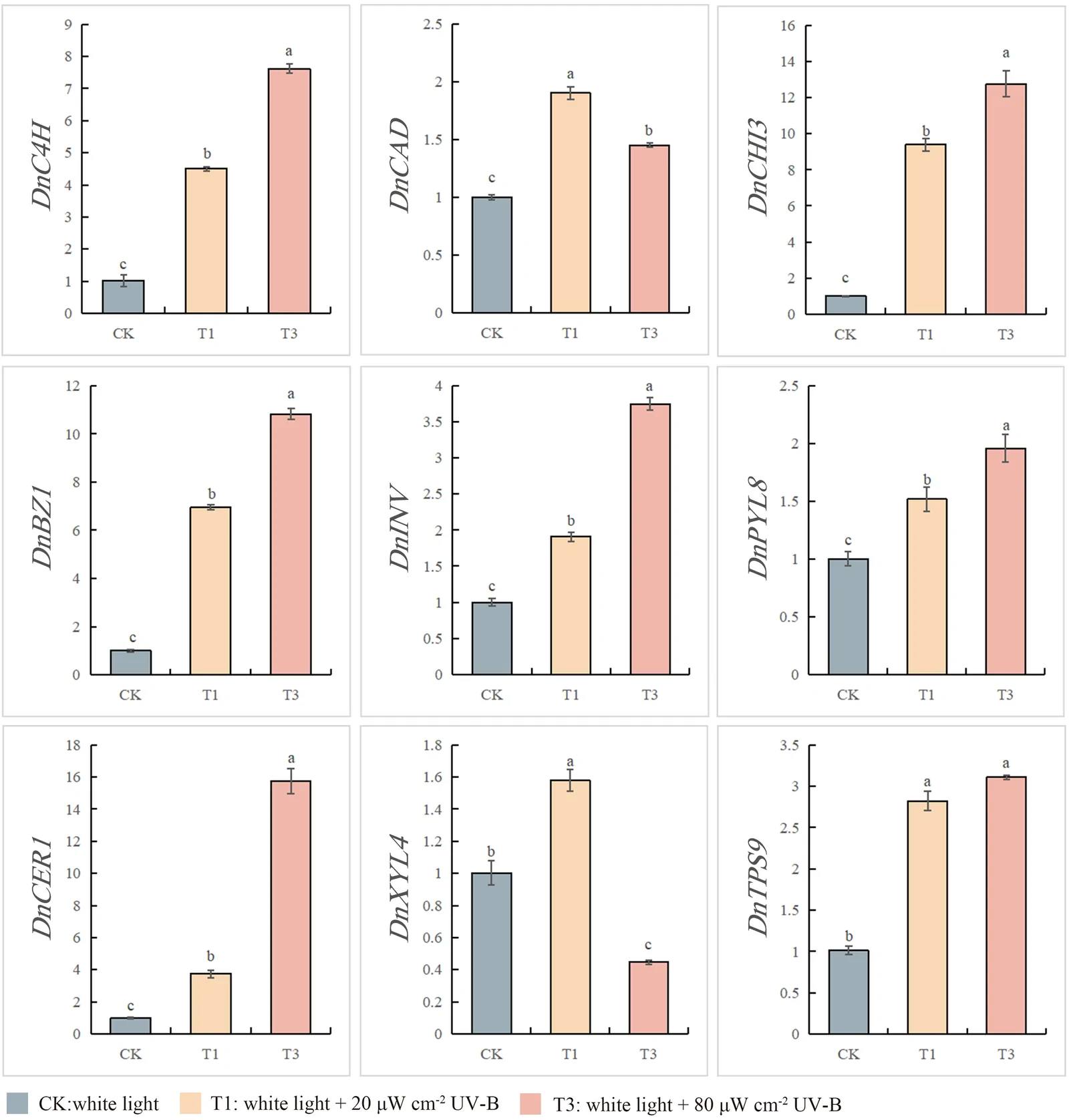
Verification of expression levels of 9 differentially expressed genes. Color codes: CG (gray), T1 (light yellow), T3 (pink).

## 4 Discussion

### 4.1 Evaluation of UV-B acclimation regulation and stress response in *D. nobile*

UV-B induces acclimation responses and activates additional resistance mechanisms with increasing intensity (Chen et al., 2022). In *Dendrobium nobile* Lindl., UV-B below 40 μW cm^-^² caused no detectable leaf damage, whereas 80 μW cm^-^² produced visible injury and 160 μW cm^-^² led to progressive damage with pronounced defoliation (**Figure 1a**). This is consistent with the report of Chen et al. (2023) that stress-inducing UV-B doses exceed cellular repair capacity, disrupting membranes and stomata. Osmoregulatory substances stabilize proteins and membranes while preventing dehydration (Sikder et al., 2020), with SP as a primary osmotic regulator (Wennerström and Oliveberg, 2022). Photosynthetic pigment contents increased at 40 and 80 μW cm^-2^ but decreased at 160 μW cm^-2^ (**Figures 1b–d**), with levels at 20 μW cm^-2^ statistically indistinguishable from CG (P > 0.05). At 20 μW cm^-2^, SP and ASA remained unchanged versus CG, indicating no significant osmotic stress, but both peaked at 80 μW cm^-2^, reflecting active osmoregulation. SS showed greater sensitivity, increasing at 20 μW cm^-2^, reflecting their dual osmoregulatory and signaling roles (Rosa et al., 2009). MDA content, a lipid peroxidation marker (Janero, 1990), showed no significant change at 20 μW cm^-2^ (P > 0.05) but increased at 40 and 80 μW cm^-2^, indicating progressive membrane oxidative damage. Consistent with this, PCA-based comprehensive evaluation ranked T1 highest in major chemical classes regulation and T3 highest in stress resistance. The maintenance of photosynthetic pigments and membrane integrity at 20 μW cm^-2^, together with its highest PCA score for chemical accumulation and transcriptomic signatures dominated by UVR8-mediated photomorphogenic signaling rather than general stress markers, supports the interpretation of this dose as an acclimation state. In contrast, 80 μW cm^-2^ represents a stress condition with maximal antioxidant engagement and non-specific stress signaling. *D. nobile* exhibited coordinated upregulation of CAT and SOD activities at 80 μW cm^-2^, consistent with an enhanced demand for H_2_O_2_ and O_2_^-^ scavenging (Nahar et al., 2022) in response to UV-B-induced oxidative challenge. SOD converts O_2_^-^ to H_2_O_2_ (Río et al., 2018), while CAT detoxifies H_2_O_2_ (Mhamdi et al., 2010). Transcriptomic analysis revealed MAPK pathway activation at 80 μW cm^-2^, which addresses acute UV-B stress largely independently of UVR8 (Shi and Liu, 2021), further supporting the occurrence of a systemic stress response under this condition. At 160 μμW cm^-2^, SOD/CAT decline and MDA peak indicate antioxidant system collapse rather than regulation. Although T4 transcriptomic data are unavailable, severe physiological damage suggests the tolerance threshold was exceeded, potentially involving oxidative enzyme inactivation, suppression of *de novo* biosynthesis, and disruption of cellular compartmentalization (Mittler et al., 2004). Sustained CAT activity does not explain the MDA increase, as CAT is peroxisomal and cannot compensate for cytosolic SOD-POD failure or neutralize ONOO^-^. Thus, cumulative membrane damage proceeds despite partial CAT function.

### 4.2 Effects of UV-B on photosynthetic pigments

Chlorophyll concentration reflects photosynthetic capacity, as its elevation correlates with increased ATP and NADPH production and enhanced photosynthetic rates. However, UV-B effects on photosynthetic pigments remain controversial, with reports of both increased and decreased chlorophyll content (DeLong and Steffen, 1997; Musilf et al., 2002; Jovanić et al., 2022). In this study, chlorophyll a and b in *D. nobile* leaves increased at UV-B up to 80 μW cm^-^² but decreased at 160 μW cm^-^² (**Figure 1**).

Chlorophyll synthesis requires phytyl-PP, which derives from GGPP via geranylgeranyl reductase (GGR), encoded by *chlP* (Karlický et al., 2021). Transcriptomic analysis revealed significant downregulation of *DnchlP* (*DnGGR*) under 20 and 80 μW cm^-^² UV-B, which would be expected to reduce phytyl-PP supply and thus *de novo* synthesis (Tanaka et al., 1999). Nevertheless, chlorophyll content increased at these intensities, likely due to the significant downregulation of *STAYGREEN* (*SGR*), a key gene encoding Mg-dechelatase that initiates chlorophyll degradation (Hörtensteiner and Kräutler, 2011; Hu et al., 2021). Consistent with Wang et al. (2022a), *SGR* downregulation suppresses leaf discoloration and stabilizes chlorophyll content. Thus, we propose that UV-B did not promote chlorophyll synthesis in *D. nobile*, but rather led to net chlorophyll accumulation through attenuated degradation.

Modulating *SGR* expression has agricultural relevance: *OsSGR* manipulation delays senescence and enhances yield in rice (Shin et al., 2020), while *CsSGR* downregulation confers cold tolerance in cucumber (Dong et al., 2023). Consistent with these findings, inhibiting chlorophyll degradation can delay senescence (Tanaka and Ito, 2025). In this study, 20 μW cm^-^² UV-B significantly downregulated *DnSGR*, effectively controlling chlorophyll degradation, enhancing stay-green trait, and potentially improving stress tolerance, biomass, and quality in *D. nobile*.

### 4.3 UV-B modulates a broad transcription factor network for growth acclimation

Plant adaptation to UV-B is key to achieving UV-B tolerance, and the UVR8 photoreceptor signaling pathway plays a central role in this process. Upon sensing UV-B radiation, UVR8 dimers dissociate into monomers, translocate into the nucleus, and interact directly or indirectly with multiple transcription factors, thereby activating downstream signaling cascades (Christie et al., 2012). Transcriptomic data reveal that the expression levels of signaling-related genes, including *DnUVR8*, *DnSPA1*, *DnCOP1*, and *DnRUP2*, increase with rising UV-B intensity. This coordinated regulatory strategy simultaneously enhances signal perception and transduction capacity (via upregulation of UVR8 and COP1/SPA1) while reinforcing negative feedback loops to avert excessive stress responses (via upregulation of RUP2).

Under low-intensity UV-B radiation, the expression of multiple transcription factors involved in growth and development is significantly altered. Downregulated factors include *DnPCL1 (DnLUX)*, *DnMYB2-l*, *DnMYBx2*, *DnERF9-l*, *DnERF5-l*, *DnBHLH74*, *DnBIM2*, *DnWRKY46*, *DnWRKY75*, *DnSCL7-l*, and *DnSCL9-l*. The downregulation of *PCL1* would be expected to relieve repression of *PIF4* and *PIF5*, potentially promoting hypocotyl elongation (Nusinow et al., 2011). Nevertheless, UV-B-induced hypocotyl growth is clearly inhibited, suggesting that the UVR8 pathway may override the circadian clock program by tagging *PIF4* and *PIF5* for *COP1*-mediated proteasomal degradation, thereby contributing to growth suppression (Tavridou et al., 2020; Sharma et al., 2019a). *MYBx1*, a homolog of *MYBx2*, acts as a repressor of anthocyanin biosynthesis by blocking transcriptional activation of target genes such as *DFR* and *ANS* (Fu et al., 2019). In our experiments, the significant downregulation of *DnMYBx2*, accompanied by upregulation of *DnANS*, suggests that this regulatory module may also be conserved in UV-B responses, positioning *DnMYBx2* as a candidate negative regulator of anthocyanin accumulation under UV-B. *BHLH74*, a positive regulator of cell elongation (Bao et al., 2014), may contribute to UV-B-induced hypocotyl growth inhibition via its downregulation and thus represents a candidate mediator of growth suppression. Moreover, UVR8 can directly interact with BIM1 and BES1 to suppress their DNA-binding activity (Liang et al., 2018), thereby restraining hypocotyl elongation, and BIM2 may function at a similar integration point between brassinosteroid (BR) and light signaling. Finally, *SCL9-l*, a GRAS family member, emerges as a candidate node potentially linking light, hormonal, and stress response pathways (Li et al., 2019).

Upregulated genes include *DnHY5, DnMYBS3*, *DnRL3*, *DnBHLH66-l*, *DnWRKY2*, *DnHAT5*, *DnTCP7*, *DnPCF2*, *DnHSFC-2B*, *DnCAL1*, *DnC3H2*, *DnC3H14*, and *DnSPL18*. Among these, HY5 is stabilized via UVR8-COP1 interaction, leading to its accumulation. As a central regulator of UV-B-induced photomorphogenesis, HY5 directly activates numerous UV-B-responsive genes by binding to their promoters, thereby triggering downstream responses including hypocotyl growth suppression and accumulation of photoprotective pigments such as flavonoids and anthocyanins (Huang et al., 2013; Binkert et al., 2014). Consistent with this known role, *DnHY5* is upregulated in our dataset and may serve as a master hub connecting UV-B perception to transcriptional reprogramming for photomorphogenic adaptation. *MYBS3* overexpression reduces plant height (Li et al., 2026), suggesting that *DnMYBS3* is a candidate regulator potentially prioritizing adaptive responses over elongation. *BHLH66-l*, associated with chlorophyll and carotenoid metabolism and root hair development (Luo et al., 2022; Bruex et al., 2012), may coordinate photosynthetic pigment homeostasis and root architecture under UV-B and is therefore identified as a candidate acclimation-associated factor. *WRKY2* helps maintain circadian *PIF4* expression via CCA1/LHY (Wang et al., 2022c). This transcription factor may integrate UV signaling with the circadian clock to optimize growth-acclimation balance, making it a candidate integrator of these pathways. *PCF2* affects plant height, root growth, and stomatal regulation (Almeida et al., 2017), potentially contributing to morphological adjustments that limit water loss under UV-B. *ZF CCCH2* (*C3H2*) has been reported to promote germination and lateral root development (He et al., 2022), which may enhance root plasticity to sustain nutrient uptake during UV-B acclimation. In contrast, *ZF CCCH14* (*AtC3H14*) negatively regulates growth and cell elongation via transcriptional and RNA-binding activities (Kim et al., 2014); Co-upregulation of *DnC3H14* and *DnC3H2* points to a coordinated recruitment of growth-promoting and -restraining factors, possibly to optimize the growth-acclimation balance.

Based on their differential expression under low-intensity UV-B, these transcription factors are proposed as candidate regulators of photomorphogenic acclimation that warrant functional validation. In contrast, when UV-B intensity reaches 80 μW cm^-^², additional transcription factors, including *DnMYBC1*, *DnODO1*, *DnMYB12*, and *DnMYB41*, are recruited into the regulatory network. These factors may function primarily in damage repair and defense-related processes, suggesting a transition in the plant’s regulatory strategy from active acclimation to passive protection.

### 4.4 Mechanisms of UV-B acclimation regulation of major chemical classes in *D. nobile.*

Polyphenols play a protective role against external stressors (Rana et al., 2022). UV-B at 20 μW cm^-^² significantly enhanced polyphenol accumulation in *D. nobile* by 41.0% over the CG, accompanied by marked upregulation of *DnC4H*, *DnCAD*, and *DnPOD12*. Cinnamic acid is converted to p-coumaric acid by cinnamic acid 4-hydroxylase (C4H). The resulting p-coumaric acid then enters the monolignol biosynthesis pathway, a process involving cinnamyl alcohol dehydrogenase (CAD), ultimately leading to lignin polymerization by peroxidases (Sharma et al., 2019b; Wang et al., 2022b). UV-B significantly regulated *CAD* expression, consistent with observations in *Allium cepa* L (Widiastuti et al., 2024). CAD, a key enzyme in monolignol biosynthesis, is required for polyphenol production and flavonoid precursors. Similarly, UV-B upregulated *GhC4H* expression in *Gossypium hirsutum* L., promoting phenolic accumulation and UV-B tolerance (Song et al., 2025). Peroxidase catalyzes lignin monomer polymerization, completing lignin biosynthesis (Xiao et al., 2023). However, unlike these sun-exposed crops, *D. nobile* is an epiphytic orchid naturally shaded by the forest canopy and adapted to low-UV environments, thus likely exhibiting a lower UV-B tolerance threshold. In this study, compared to T1, upstream *DnC4H* remained upregulated at T3, but the expression of downstream *DnPOD12* and *DnCAD* was selectively suppressed by high-dose UV-B. This decoupling between upstream activation and downstream suppression reflects distinct sensitivity thresholds among different metabolic nodes in response to escalating stress. Consequently, only under the acclimation-triggering dose (T1) were phenylpropanoid pathway genes coordinately upregulated to effectively promote phenolic accumulation, whereas the high-dose treatment (T3) disrupted the continuity of downstream biosynthesis. In addition, under high UV-B stress, limited carbon resources may be preferentially allocated to flavonoid biosynthesis, which produces more potent UV-absorbing compounds, at the expense of polyphenol accumulation, reflecting a resource reallocation strategy that further contributes to their divergent accumulation patterns. The differential sensitivity observed here thus reflects not only the inherent regulatory capacity of the plant but also its evolutionary adaptation to a low-UV microenvironment.

UV-B promotes flavonoid accumulation in plants (Xie et al., 2022; Neugart and Bumke-Vogt, 2021). Exposure to 20 μW cm^-^² UV-B enhanced flavonoid accumulation by 16.3% in *D. nobile* over the CG, accompanied by marked upregulation of the *DnCHS*, *DnCHI*, and *DnCHI3*. Flavonoid biosynthesis involves a dynamic process mediated by multiple enzymes including phenylalanine ammonia-lyase (PAL), cinnamate-4-hydroxylase (C4H), 4-coumaroyl-CoA ligase (4CL), chalcone synthase (CHS), chalcone isomerase (CHI), flavanone synthase (FLS), and flavonoid 3’-hydroxylase (F3’H) (Zhao et al., 2020a). Flavonoid synthesis critically depends on *CHI* and *CHS*, with deficient plants exhibiting substantially reduced production (Lam et al., 2022; Ma et al., 2023). Previous studies demonstrated that UV-B triggers UVR8 interaction with MYB13 and HY5 transcription factors to activate *CHS* and *CHI*, promoting flavonoid biosynthesis (Binkert et al., 2014). The *DnBZ1*, associated with anthocyanin biosynthesis, also exhibited significant upregulation. This gene encodes anthocyanin 3-O-glucosyltransferase, a key enzyme in the anthocyanin biosynthetic pathway, and participates in reactive oxygen species scavenging (Wang et al., 2022c). Compared with 20 μW cm^-2^ UV-B, the 80 μW cm^-2^ treatment showed stronger upregulation of *DnCHS*, *DnCHI*, *DnBZ1*, and *DnCHI3*. High-intensity UV-B also activated elevated expression of transcription factors *DnMYB12*, *DnMYB41*, and *DnMYBC1*. MYB12 acts as a flavanol-specific regulator and transcriptional activator of chalcone synthase and flavonol synthase (Mehrtens et al., 2005). Overexpression of *MYB41* and *MYBC1* induces *PAL* and *F3’H* expression, respectively, enhancing flavonoid and proanthocyanidin accumulation (Jin et al., 2024; Peng et al., 2020). Notably, flavonoid content continued to increase up to 160 μW cm^-2^ (T4), reaching the highest level among all treatments. This sustained accumulation, even under severe physiological deterioration, suggests that flavonoid biosynthesis operates under a distinct regulatory logic compared to polyphenols: flavonoid biosynthetic genes and their regulators remained highly responsive to UV-B intensity, whereas polyphenol pathway genes were suppressed at high doses. This indicates that flavonoid accumulation is UV-B dose-dependently regulated even under extreme stress.

Dendrobine, the predominant alkaloid in *D. nobile*, exhibits significant pharmacological effects (Song et al., 2019; Silva and Ng, 2017). At 20 μW cm^-2^, UV-B significantly increased alkaloid content by 33.1% over CG, consistent with low-intensity UV-B promoting alkaloid accumulation in *D. officinale* (Chen et al., 2020b). Transcriptome analysis revealed upregulation of MVA pathway genes *DnMVD* and *DnMVK*, key candidates for dendrobine biosynthesis (Li et al., 2017; Wang et al., 2021b), along with terpene synthase *DnTPS9*, a known regulator of terpenoid accumulation in Dendrobium (Han et al., 2024). At T3 (80 μW cm^-2^), however, alkaloid content did not increase significantly despite the sustained upregulation of *DnMVD* and *DnTPS9*. Notably, the expression of *DnMVK*, a rate-limiting kinase in the MVA pathway, was significantly downregulated at T3 compared to T1. Given that MVK catalyzes the phosphorylation of mevalonate-5-phosphate, a committed step in isoprenoid precursor supply, its suppression likely creates a metabolic bottleneck that restricts downstream flux toward dendrobine skeleton formation, even when upstream (*DnMVD*) and downstream (*DnTPS9*) genes remain transcriptionally active. This differential response, where *DnMVK* exhibits a lower activation threshold and higher sensitivity to elevated UV-B intensity than *DnMVD* or *DnTPS9*, suggests that high-dose UV-B imposes branch-specific regulatory constraints on the MVA pathway, rather than uniformly suppressing terpenoid biosynthesis. Collectively, these results confirm that low-intensity UV-B (T1) effectively induces alkaloid accumulation in *D. nobile*, whereas the high-intensity treatment (T3) disrupts the coordinated precursor supply required for sustained dendrobine biosynthesis.

Dendrobium polysaccharides accumulate under appropriate UV-B exposure as a cellular defense mechanism (Cui et al., 2020; Wang et al., 2018). In this study, 20 μW cm^-^² UV-B increased polysaccharide content by 19.3%. Transcriptomic analysis revealed significant enrichment of DEGs in galactose, fructose/mannose, and starch/sucrose metabolism. Sucrose invertase (INV) hydrolyzes sucrose to glucose and fructose, providing energy and substrates (Ruan, 2012). *DnMAN9*, a *mannan endo-1,4-beta-mannosidase* (*MAN*) gene in mannose metabolism, was significantly upregulated, and enhanced *MAN* expression stimulates production of primary active constituents (Niu et al., 2021; Hu et al., 2019). In galactose metabolism, *DngalM* (*galactose mutarotase*), which catalyzes the initial step converting galactose to α-D-galactose, was significantly downregulated, potentially promoting D-galactose accumulation (Lu et al., 2021; Han et al., 2023). Additionally, *DnTPP* (*trehalose-6-phosphate phosphatase*) participates in trehalose biosynthesis, which regulates plant growth and stress responses through the precursor signaling molecule trehalose-6-phosphate, while trehalose itself acts as an osmoprotectant (Fernandez et al., 2010). And GolS (galactinol synthase), a key enzyme in raffinose family oligosaccharide biosynthesis functioning as an osmoprotectant, were both significantly upregulated (Nishizawa et al., 2008). Collectively, UV-B modulates multiple sugar metabolic pathways to enhance polysaccharide content in *D. nobile* at 20 μW cm^-^². At T4, polysaccharides showed substantial accumulation, which may be attributed to severe UV-B stress (160 μW cm^-^²) that inhibited plant growth and restricted carbon sinks, redirecting carbon flux from structural consumption toward polysaccharide accumulation (Pallas et al., 2013). The surge in polysaccharides at T4 represents a passive terminal accumulation driven by carbon sink limitation, rather than the active synthesis observed at T1/T3.

### 4.5 Defense mechanisms in response to UV-B stress of *D. nobile*

At UV-B intensities up to 80 μW cm^-2^, *D. nobile* exhibited coordinated upregulation of antioxidant enzymes, osmoregulatory substances, and flavonoids (**Figure 1**). GO and KEGG enrichment analyses showed DEGs enrichment in abiotic stress, light stress, and radiation response processes versus CG. Genes associated with MAPK signaling and cell wall biosynthesis also demonstrated significant regulation. Transcription factor profiling revealed substantial regulation of MYB family members, which regulate diverse biological processes including growth, development, phytohormone signaling, secondary metabolism, and abiotic stress responses (Ma et al., 2025). Among these, *MYB12* and *MYB41* promote flavonoid biosynthesis and enhance stress tolerance. *MbMYBC1* upregulation also activates *AtSnRK* expression in *Arabidopsis* and improves resistance (Liu et al., 2023a). Compared to T1 and CG, *DnMYB12*, *DnMYB41*, and *DnMYBC1* were all significantly upregulated, suggesting these orthologs may regulate stress responses intensified by elevated UV-B. *SiMYBS3* enhances stress tolerance in *S. italica* (Liu et al., 2023b), while overexpression of *PtrbHLH66* reduces MDA accumulation and alleviates oxidative damage (Liang et al., 2022).

High-intensity UV-B also activated abscisic acid (ABA) signaling. As a stress-induced phytohormone, ABA acts as a long-distance signal for oxidative damage, triggering defense responses and stomatal regulation via the MAPK cascade (Ko and Helariutta, 2017). Under 80 μW cm^-^² UV-B, *DnSnRK2*, *DnMAPKKK17*, *DnPYL3*, and *DnPYL8* were upregulated. Signaling initiates when PYL receptors bind ABA and inhibit PP2C phosphatases, which releases SnRK2 kinases to transduce stress signals (Chen et al., 2020a). The subsequent SnRK2-mediated activation of MAPKKK17 initiates a phosphorylation cascade (Kong et al., 2020), enhancing oxidase activity and promoting stomatal closure to reduce CO_2_ fixation (Li et al., 2022).

Xyloglucan endotransglycosylase/hydrolase genes *DnXTH2*, *DnXTH9*, *DnXTH28*, *DnXTH23*, and *DnXTH32* were significantly regulated under UV-B stress. *XTH* play crucial roles in abiotic stress resistance by modifying cellulose-xyloglucan complex structure, and *XTH9* upregulation stimulates xylem production and secondary wall thickening (Kushwah et al., 2020). UV-B stress also upregulated *DnCYP77A6* and *DnCER1* regulating cuticle synthesis. The cuticle constitutes a specialized lipid cell wall modification, a defensive barrier of waxes and cutin that prevents water loss and reduces UV-B penetration (Yeats and Rose, 2013). This aligns with findings that UV-B increases cell wall and cuticle thickness (Laakso et al., 2000). Flavonoids function as natural UV-absorbing sunscreens, reducing oxidative damage (Nunes et al., 2018). Under UV-B stress, flavonoid synthesis genes, *DnCHI*, *DnC4H*, *DnCHS*, and *DnBZ1* showed stronger upregulation than under mild UV-B.

UV-B stress also causes DNA damage. Direct DNA absorption of UV-B induces cyclobutane pyrimidine dimers (CPD) and pyrimidine (6-4) pyrimidone photoproducts (6-4PPs), which disrupt base pairing and block DNA replication and transcription, potentially leading to gene mutations (Qüesta et al., 2013). CPD photolyase encoded by the *PHR* repairs CPD lesions in a UVR8-dependent manner and alleviating UV-B-induced growth inhibition (Li et al., 2015). Upregulation of the *DASH-type cryptochro*me (*Cry-DASH*) mediates the repair of UV-B-induced CPDs (Kiontke et al., 2020). The *(6-4) DNA photolyase* (*H64PHR*) is similarly significantly up-regulated under UV-B stress and repairs UV-B-induced DNA damage (Faraji and Dreuw, 2014).

### 4.6 The effect of UV-B management on the cultivation of *D. nobile*

Integration of physiological indicators and principal component analysis (**Table 1**) showed that all UV-B doses increased major chemical classes content though the response patterns differed across compound classes and treatment doses. Among the treatments, T1 demonstrated the highest overall major chemical classes content. The 20 μW cm^-^² UV-B treatment exhibited higher acclimation capacity. Compared to CG, the 20 μW cm^-^² UV-B treatment significantly increased flavonoid, polyphenol and alkaloid contents by 16.3%, 41.0%, and 33.1%, respectively. *D. nobile* exposed to 80 μW cm^-^² UV-B exhibited pronounced physiological stress, yet showed enhanced flavonoid biosynthesis with a 23.7% content increase versus CG. Continuous irradiation at 160 μW cm^-^² induced irreversible stress damage. Despite this, polysaccharide and flavonoid contents were significantly elevated by 134.0% and 41.7%, respectively, compared to the CG. Flavonoids represent key secondary metabolites involved in plant UV-B resistance and exhibit a significant correlation with UV-B intensity. Consistent with the findings of Zhao et al. (2020a) in *G. biloba*, UV-B intensities that markedly increased MDA content also triggered significant flavonoid accumulation. The increase in polysaccharides may result from growth arrest and carbon sink disruption under severe stress conditions, leading to passive terminal accumulation rather than active biosynthesis. In summary, *D. nobile* exhibits high adaptability to continuous 20 μW cm^-^² UV-B, which effectively promotes polyphenol, flavonoid, and alkaloid accumulation. During the harvest period, we propose that brief exposure to high-intensity UV-B at 80 or 160 μW cm^-^² may further enhance the accumulation of flavonoids or polysaccharides.

Based on the integrated physiological, biochemical, and transcriptomic evidence, we propose a working model for UV-B-mediated regulation in *D. nobile* (**Figure 10**). Under low-intensity UV-B (20 μW cm^-2^), the UVR8 signaling module is activated, upregulating photomorphogenesis-associated transcription factors, thereby promoting secondary metabolism without oxidative damage. By contrast, high-intensity UV-B (80 μW cm^-2^) engages stress-responsive transcription factors via MAPK pathway activation, coinciding with the induction of non-specific stress responses. This model is proposed as a hypothetical framework for optimizing UV-B regimes in medicinal plant cultivation and should be interpreted as such, pending experimental validation.

**Figure 10:**
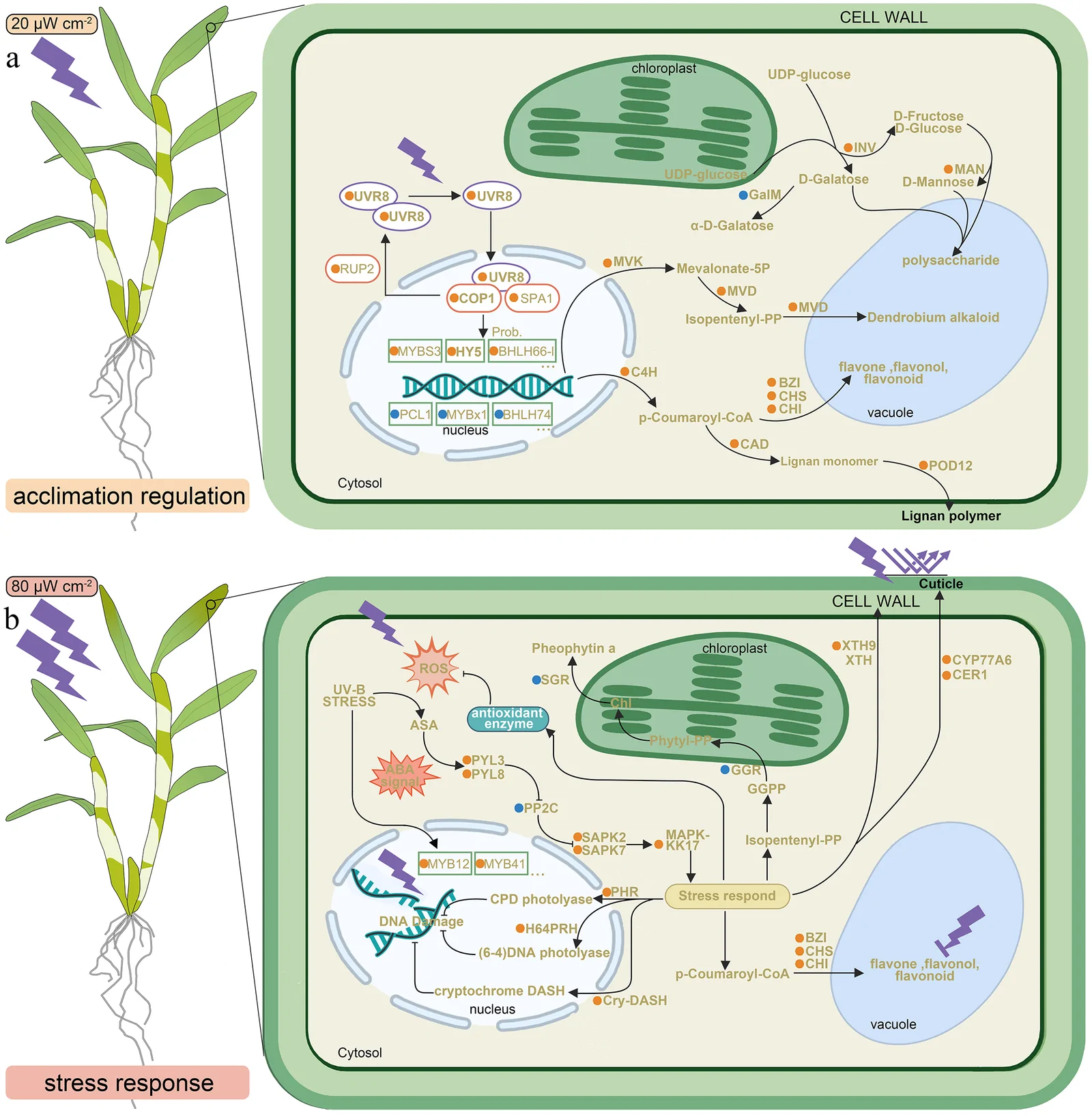
Regulatory mechanisms of UV-B irradiation on *Dendrobium nobile* Lindl. Effect of UV-B acclimation regulation on the major chemical classes **(a)**. Defense mechanisms in response to UV-B stress in *D. nobile* **(b)**.

## 5 Conclusion

This study reveals the regulatory mechanisms of *Dendrobium nobile* Lindl. in response to UV-B acclimation and stress. Within the experimental gradient, the acclimation-enhancing intensity at 20 μW cm^-^² UV-B promoted major chemical classes accumulation while suppressing chlorophyll degradation via *DnSGR* downregulation. Key biosynthetic genes in phenylpropanoid (*DnC4H*, *DnCAD*, *DnPOD12*), flavonoid (*DnCHI*, *DnCHI3*, *DnCHS*, *DnBZ1*), dendrobine (*DnMVK*, *DnMVD*, *DnTPS9*), and saccharide pathways were significantly upregulated, enhancing polysaccharide content. Several transcription factors (*DnHY5*, *DnPCL1*, *DnMYBx1*, *DnBIM2*, *DnSCL9-l*, *DnMYBS3*, *DnBHLH66-l*, *DnWRKY2*, *DnPCF2*, *DnC3H2*, *DnC3H14*) showed expression changes under adaptive UV-B, suggesting their involvement in photomorphogenic acclimation. Under elevated UV-B stress, ABA-MAPK signaling upregulated the expression/activity of antioxidant enzymes (SOD, CAT), while increased SP, SS, and ASA maintained osmotic balance. Defense barriers were reinforced via *DnXTH*, *DnCYP77A6*, and *DnCER1*-mediated cell wall and lipid modifications. Flavonoid synthesis genes were substantially upregulated to enhance UV protection, and photolyase genes (*DnPHR*, *DnCry-DASH*, *DnH64PHR*) were induced for DNA repair. A distinct set of transcription factors upregulated only under high UV-B are implicated in damage repair and defense processes. Collectively, this study reveals the transcriptional networks underlying UV-B-induced major chemical classes accumulation and stress responses in *D. nobile.* For cultivation, continuous 20 μW cm^-2^ UV-B promotes major chemical classes accumulation, with brief harvest-stage irradiation at 80 or 160 μW cm^-2^ further enhancing flavonoids or polysaccharides. Future work should identify UV-B-regulated monomers via HPLC-MS/MS and validate the functions of candidate transcription factors and key biosynthetic genes through genetic approaches to establish the molecular basis for UV-B application in cultivation.

## Data Availability

The datasets presented in this study can be found in online repositories. The names of the repository/repositories and accession number(s) can be found in the article/**Supplementary Material**. The RNA-seq data have been deposited in the NCBI SRA under BioProject PRJNA1502789 (https://www.ncbi.nlm.nih.gov/bioproject/PRJNA1502789). The physiological and chemical data supporting this study are openly available in Zenodo at https://doi.org/10.5281/zenodo.17392349.
