# Supplementary material for: Mechanisms of acclimation regulation and stress response to UV-B in Dendrobium nobile Lindl
Source: Front Plant Sci. 2026 Sep 18;17:1953265. doi: 10.3389/fpls.2026.1953265 (PMC13630736; doi:10.3389/fpls.2026.1953265)
Supplement: Supplementary file 1 [file Table1.doc]

Accession	Sample Name	SPUID	Organism	Tax ID	Isolate

SAMN61985113	CK 1 CK 1	Dendrobium nobile	94219	CK_1

SAMN61985114	CK 2 CK 2	Dendrobium nobile	94219	CK_2

SAMN61985115	CK 3 CK 3	Dendrobium nobile	94219	CK_3

SAMN61985116	T1 1	T1 1	Dendrobium nobile	94219	T1_1

SAMN61985117	T1 2	T1 2	Dendrobium nobile	94219	T1_2

SAMN61985118	T1 3	T1 3	Dendrobium nobile	94219	T1_3

SAMN61985119	T3 1	T3 1	Dendrobium nobile	94219	T3_1

SAMN61985120	T3 2	T3 2	Dendrobium nobile	94219	T3_2

SAMN61985121	T3 3	T3 3	Dendrobium nobile	94219	T3_3
