# Supplementary material for: Mechanisms of acclimation regulation and stress response to UV-B in Dendrobium nobile Lindl
Source: Front Plant Sci. 2026 Sep 18;17:1953265. doi: 10.3389/fpls.2026.1953265 (PMC13630736; doi:10.3389/fpls.2026.1953265)
Supplement: Supplementary file 2 [file Table2.doc]

Object IDs and corresponding URLs:

61985113: https://www.ncbi.nlm.nih.gov/sra/61985113

61985114: https://www.ncbi.nlm.nih.gov/sra/61985114

61985115: https://www.ncbi.nlm.nih.gov/sra/61985115

61985116: https://www.ncbi.nlm.nih.gov/sra/61985116

61985117: https://www.ncbi.nlm.nih.gov/sra/61985117

61985118: https://www.ncbi.nlm.nih.gov/sra/61985118

61985119: https://www.ncbi.nlm.nih.gov/sra/61985119

61985120: https://www.ncbi.nlm.nih.gov/sra/61985120

61985121: https://www.ncbi.nlm.nih.gov/sra/61985121

61985113: https://www.ncbi.nlm.nih.gov/sra/61985113

61985114: https://www.ncbi.nlm.nih.gov/sra/61985114

61985115: https://www.ncbi.nlm.nih.gov/sra/61985115

61985116: https://www.ncbi.nlm.nih.gov/sra/61985116

61985117: https://www.ncbi.nlm.nih.gov/sra/61985117

61985118: https://www.ncbi.nlm.nih.gov/sra/61985118

61985119: https://www.ncbi.nlm.nih.gov/sra/61985119

61985120: https://www.ncbi.nlm.nih.gov/sra/61985120

61985121: https://www.ncbi.nlm.nih.gov/sra/61985121
